# Supplementary material for: Patchiness of forest landscape can predict species distribution better than abundance: the case of a forest-dwelling passerine, the short-toed treecreeper, in central Italy
Source: PeerJ. 2016 Sep 8;4:e2398. doi: 10.7717/peerj.2398 (PMC5018664; doi:10.7717/peerj.2398)
Supplement: Table S1 — Abundance was modelled as a function of environmental suitability, based on different SDMs. * p < 0.05** p < 0.01. [file peerj-04-2398-s001.docx]

|  | .5 | .55 | .6 | .65 | .7 | .75 | .8 | .85 | .9 | .95 | .975 | .99 |
| --- | --- | --- | --- | --- | --- | --- | --- | --- | --- | --- | --- | --- |
| ANN |  |  |  |  |  |  |  |  |  |  |  |  |
| i | 0,15 | 0,15 | 0,15 | 0,16 | 0,30 | 0,43 | 0,46 | 3,00 | 0,51 | -1,81 | -1,81 | 0,00 |
| slope | 0,45 | 0,45 | 0,51 | 0,52 | 0,40 | 0,25 | 0,27 | 1,89 | 0,37 | -0,52 | -0,50 | 0,22 |
| CTA |  |  |  |  |  |  |  |  |  |  |  |  |
| i | 0,15 | 0,17 | 0,22 | 0,24 | 0,29 | 0,32 | 0,36 | 0,48 | 1,96 | -1,36 | 0,53 | -0,02 |
| slope | 0,44 | 0,42 | 0,37 | 0,36 | 0,42 | 0,39 | 0,35 | 0,24 | 1,43** | -0,25 | 0,54* | 0,22 |
| FDA |  |  |  |  |  |  |  |  |  |  |  |  |
| i | -0,14 | -0,11 | -0,09 | -0,03 | -0,01 | 1,05 | 1,21 | 1,36 | 4,37 | -2,08 | -4,35 | -0,37 |
| slope | 2,18 | 2,09 | 2,09 | 2,11 | 2,13 | 2,37 | 2,41 | 2,45 | 3,15 | 1,61 | 1,36 | 2,03 |
| GBM |  |  |  |  |  |  |  |  |  |  |  |  |
| i | 0,11 | 0,11 | 0,18 | 0,25 | 0,29 | 0,44 | 0,42 | 0,40 | 1,49 | -0,16 | 0,49 | -0,01 |
| slope | 0,37* | 0,38* | 0,38* | 0,33 | 0,31 | 0,19 | 0,26** | 0,42 | 1,14** | 0,13 | 0,51 | 0,16 |
| GLM |  |  |  |  |  |  |  |  |  |  |  |  |
| i | 0,02 | 0,18 | 0,21 | 1,17 | 0,23 | 0,26 | 1,31 | 0,50 | 0,84 | 1,38 | -1,39 | -0,08 |
| slope | 0,81** | 0,60 | 0,61* | 1,05** | 0,60* | 0,62** | 1,05** | 0,25 | -0,04 | 0,87 | 0,05 | 0,48* |
| MARS |  |  |  |  |  |  |  |  |  |  |  |  |
| i | -0,17 | -0,16 | -0,14 | -0,11 | -0,10 | -0,10 | 1,27 | 0,65 | 1,09 | -0,54 | -3,96 | -0,35 |
| slope | 0,58 | 0,61 | 0,62 | 0,64 | 0,67 | 0,67 | 1,58 | 1,30 | 1,70 | 0,25 | -2,92 | 0,44 |
| ME |  |  |  |  |  |  |  |  |  |  |  |  |
| i | -0,01 | 0,01 | 0,02 | 0,03 | 0,05 | 0,09 | 0,10 | 1,36 | 0,98 | 0,34 | 0,36 | -0,20 |
| slope | 0,52 | 0,54 | 0,55 | 0,55 | 0,57 | 0,59 | 0,60 | 1,13 | -0,32 | 0,56 | 0,78 | 0,37 |
| RF |  |  |  |  |  |  |  |  |  |  |  |  |
| i | 0,24 | 0,27 | 0,28 | 0,31 | 0,31 | 0,34 | 0,35 | 1,11 | 1,86 | 0,37 | 0,55 | 0,07 |
| slope | 0,17 | 0,20 | 0,20 | 0,22 | 0,22 | 0,24 | 0,23 | 0,56 | 0,42 | -0,33 | 0,45 | 0,01 |
