# Supplementary figures and images for: Patchiness of forest landscape can predict species distribution better than abundance: the case of a forest-dwelling passerine, the short-toed treecreeper, in central Italy
Source: PeerJ. 2016 Sep 8;4:e2398. doi: 10.7717/peerj.2398 (PMC5018664; doi:10.7717/peerj.2398)

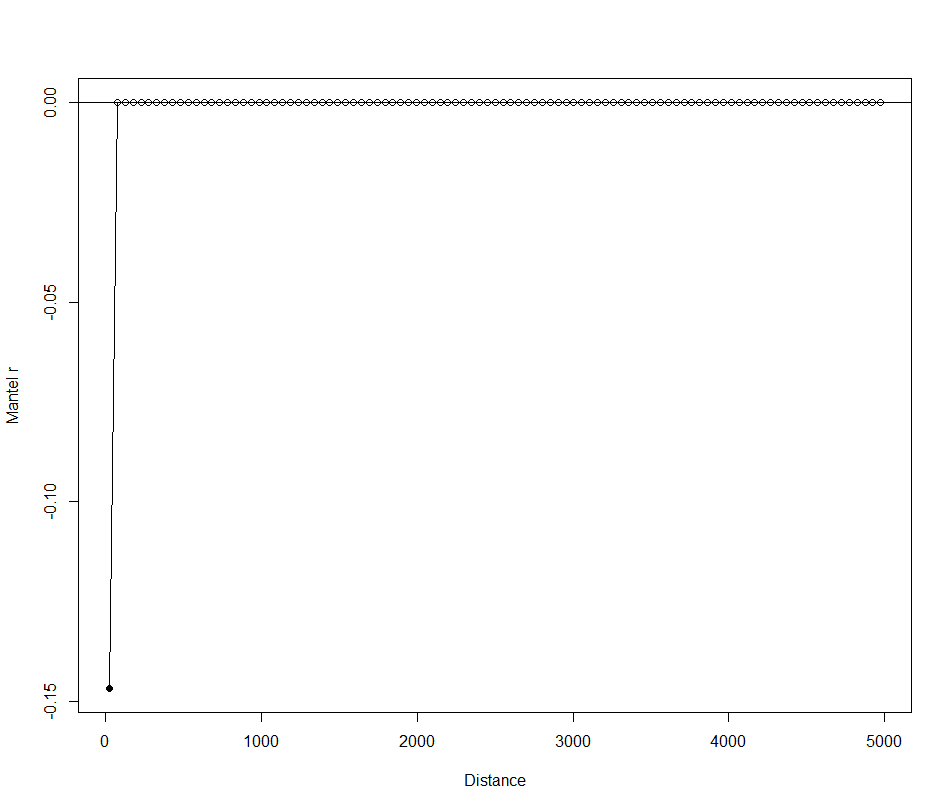

Supplement: Figure S1 — Only one significant value is present, but it is still very little. Black circle shows value significantly different from zero. White circles show non significant correlation. [file peerj-04-2398-s002.png]

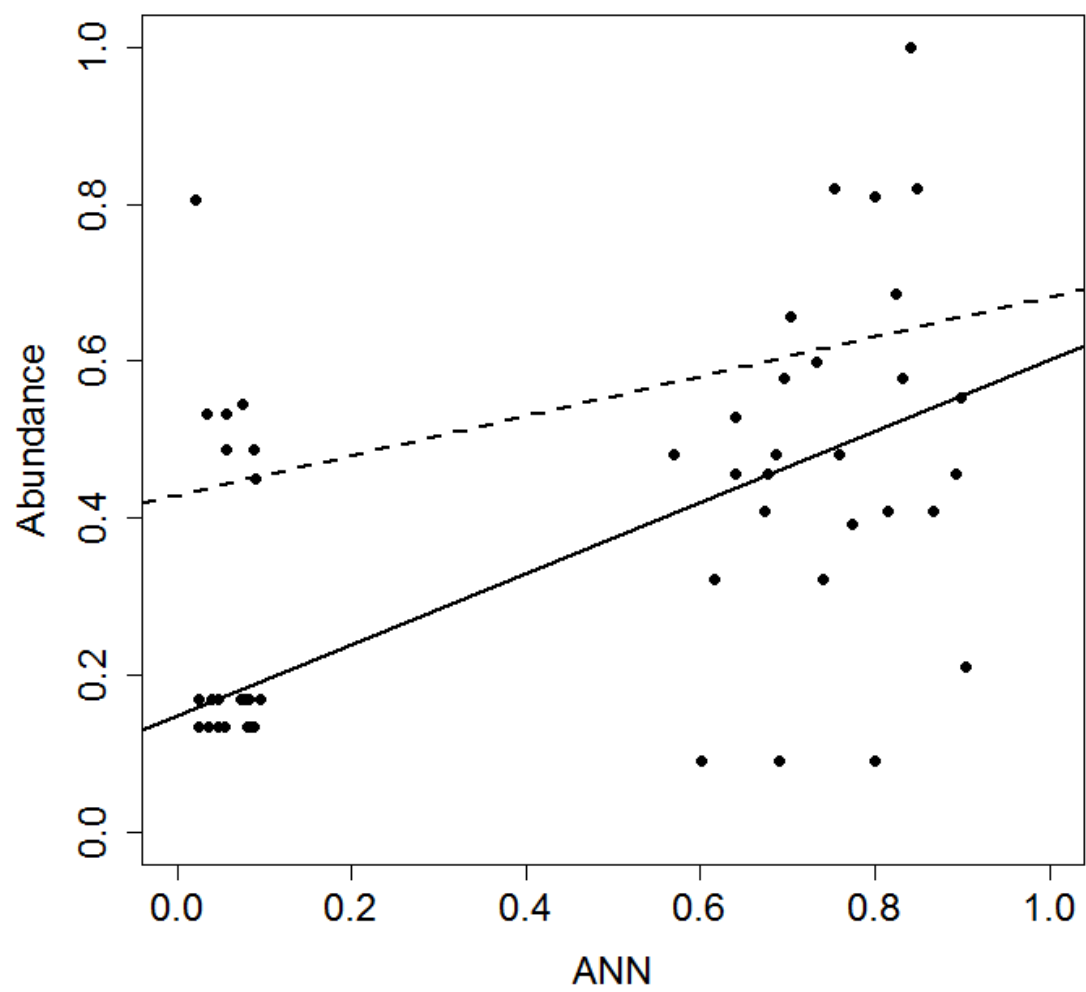

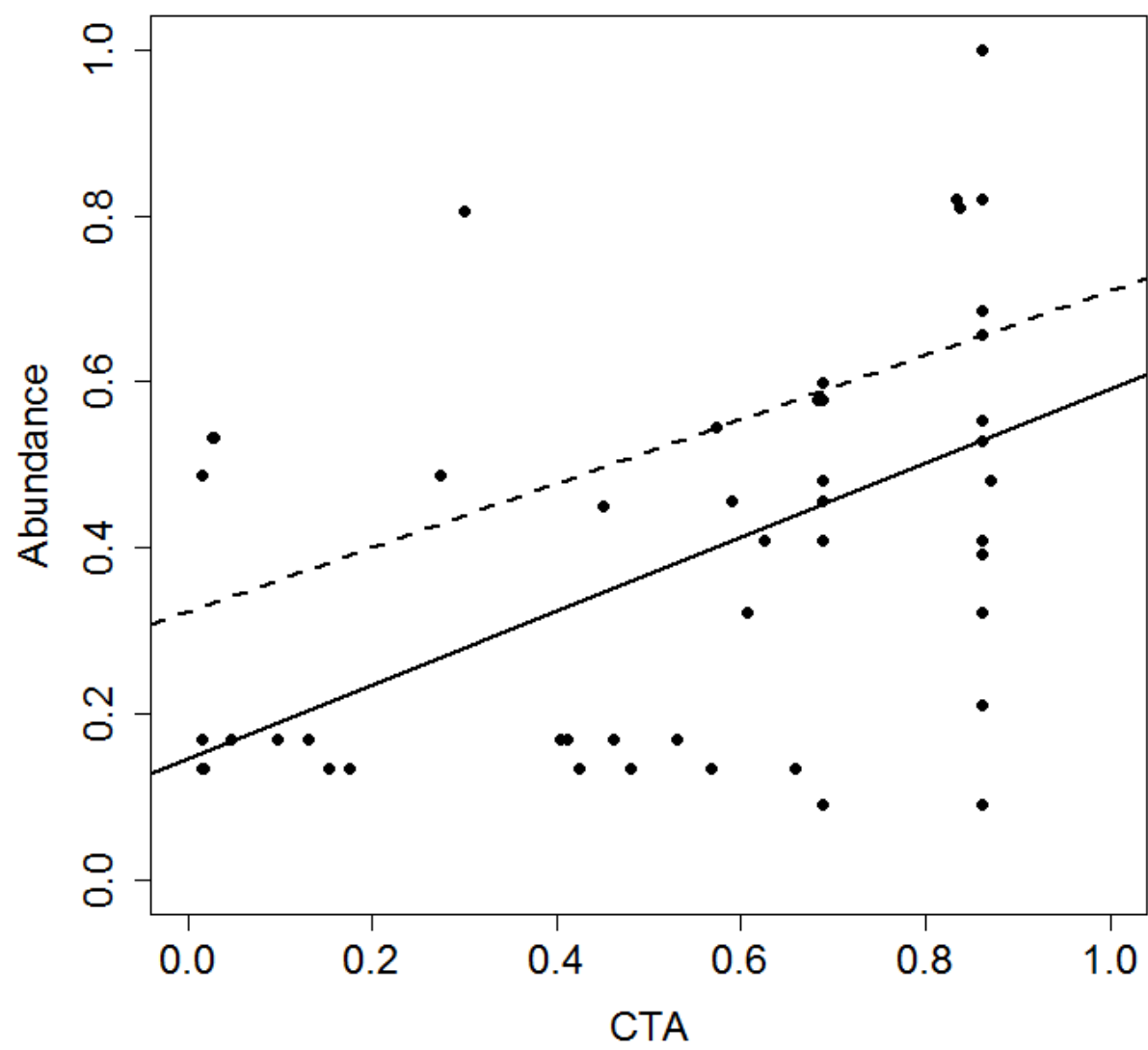

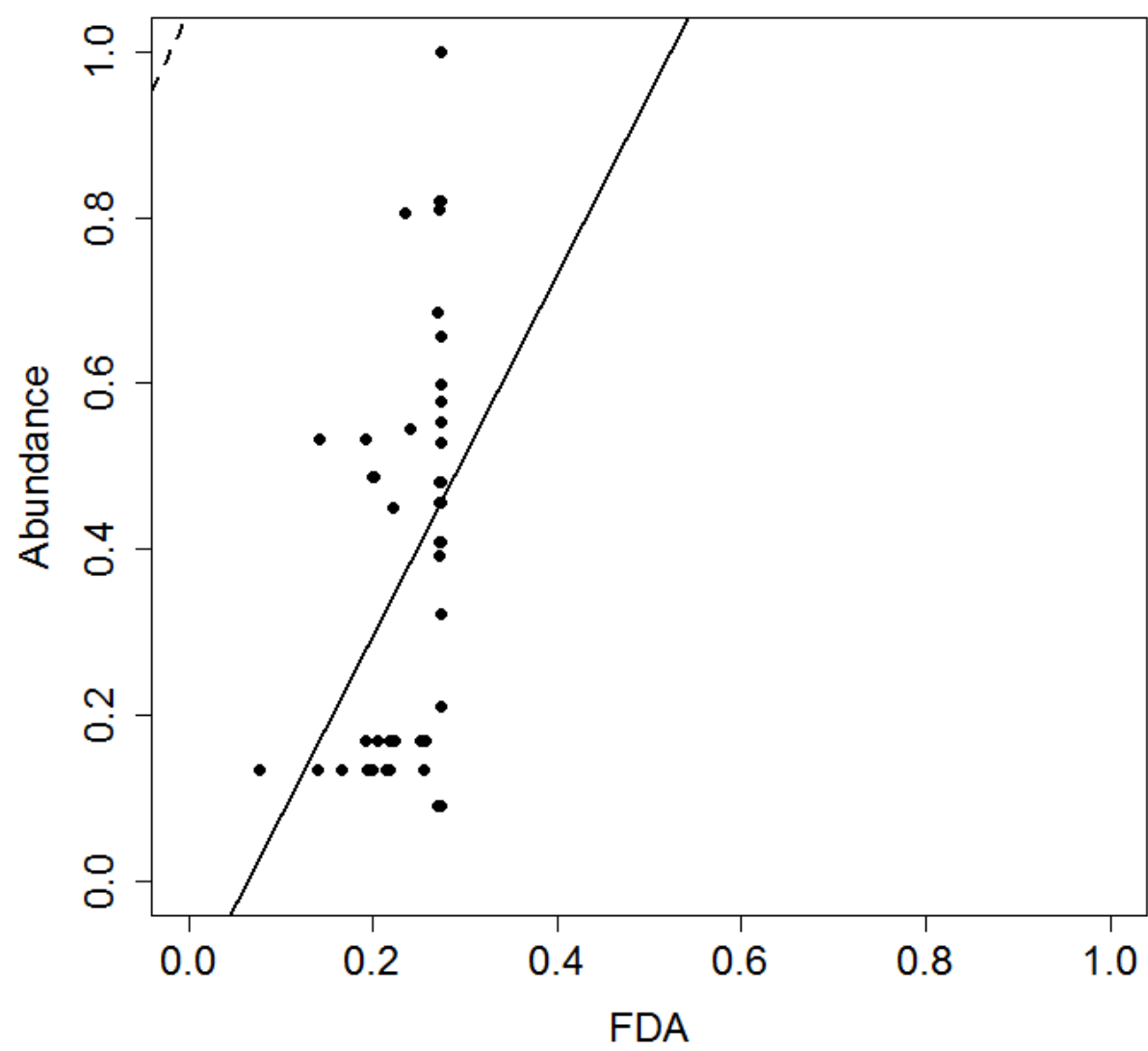

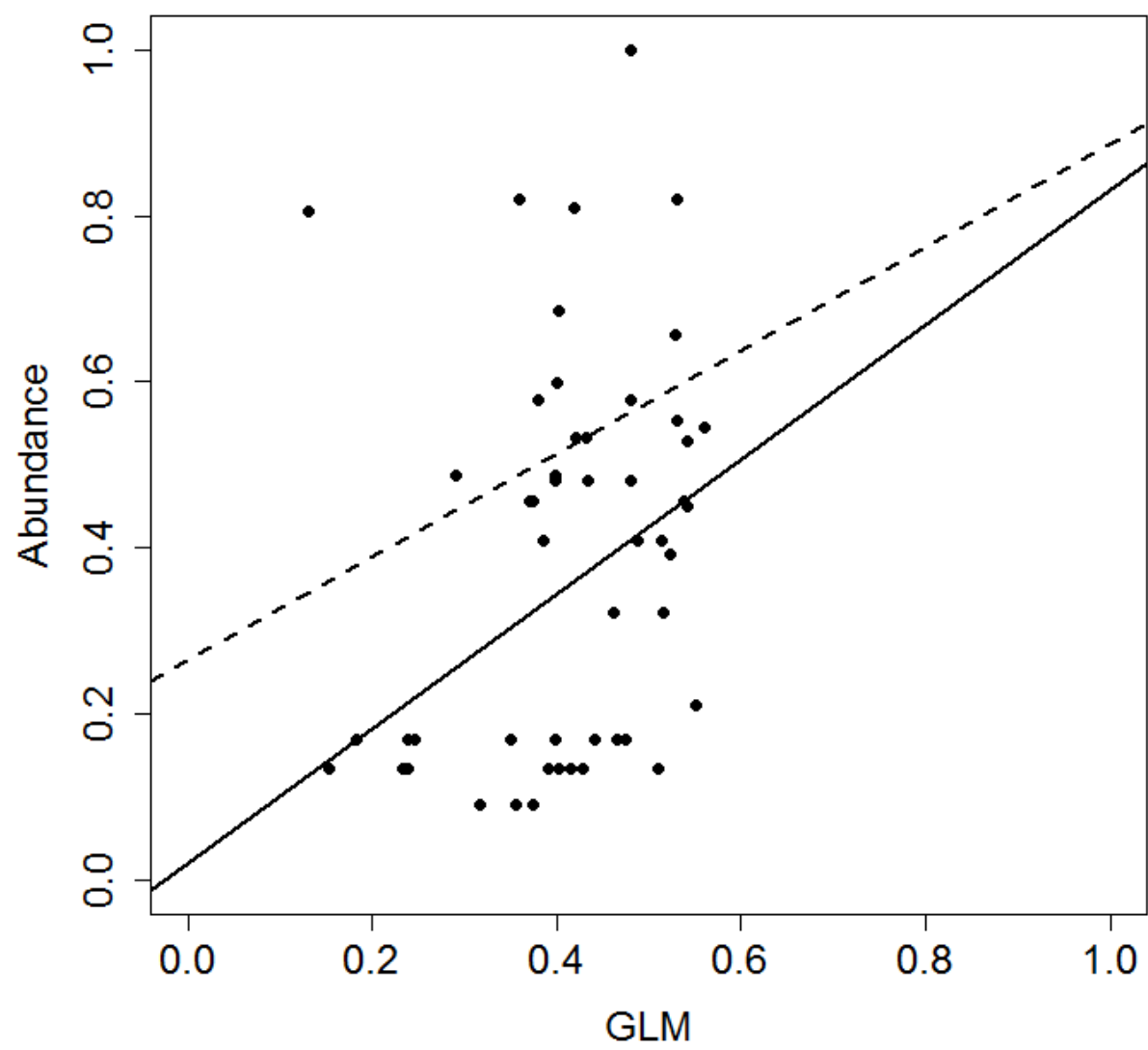

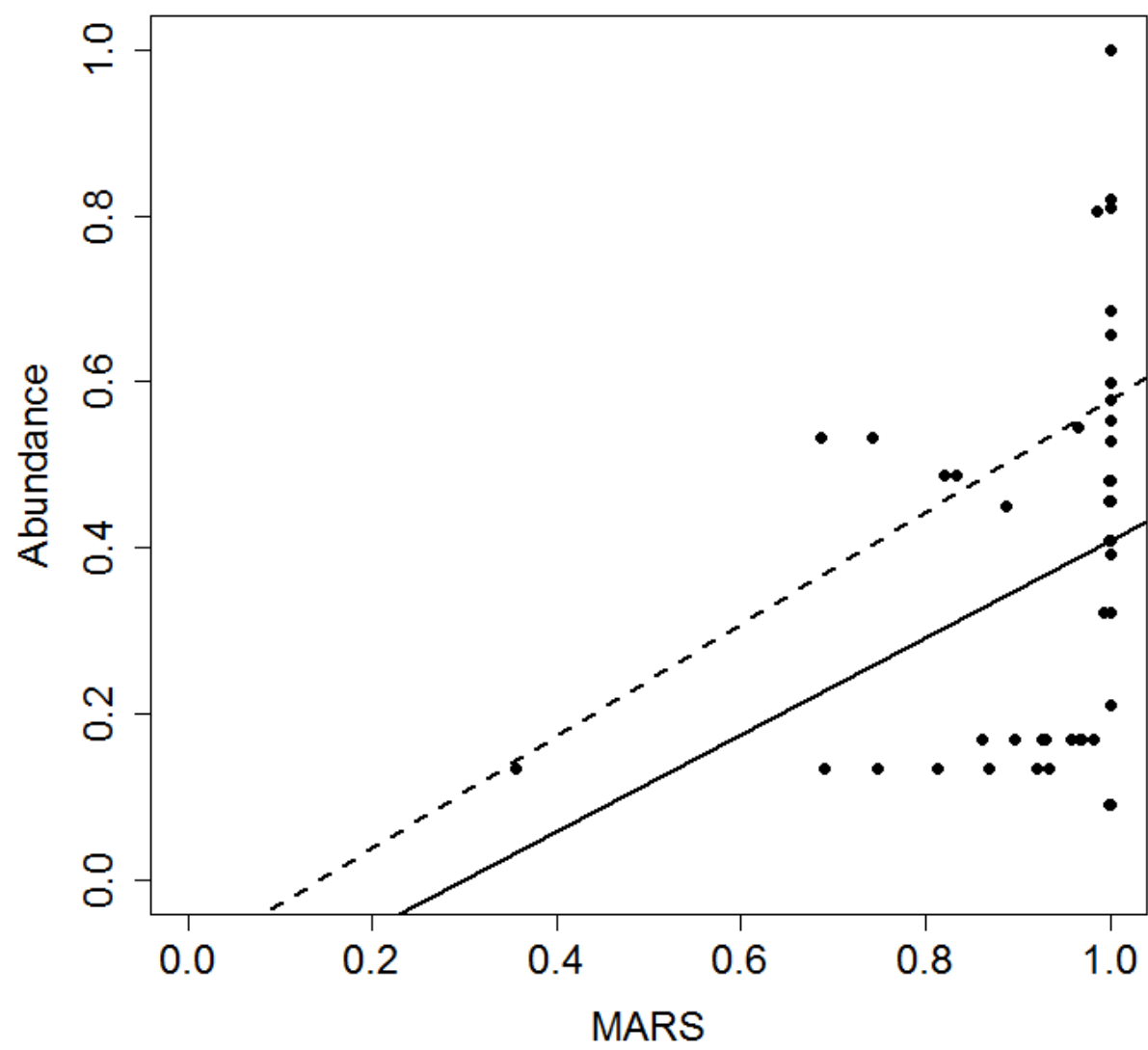

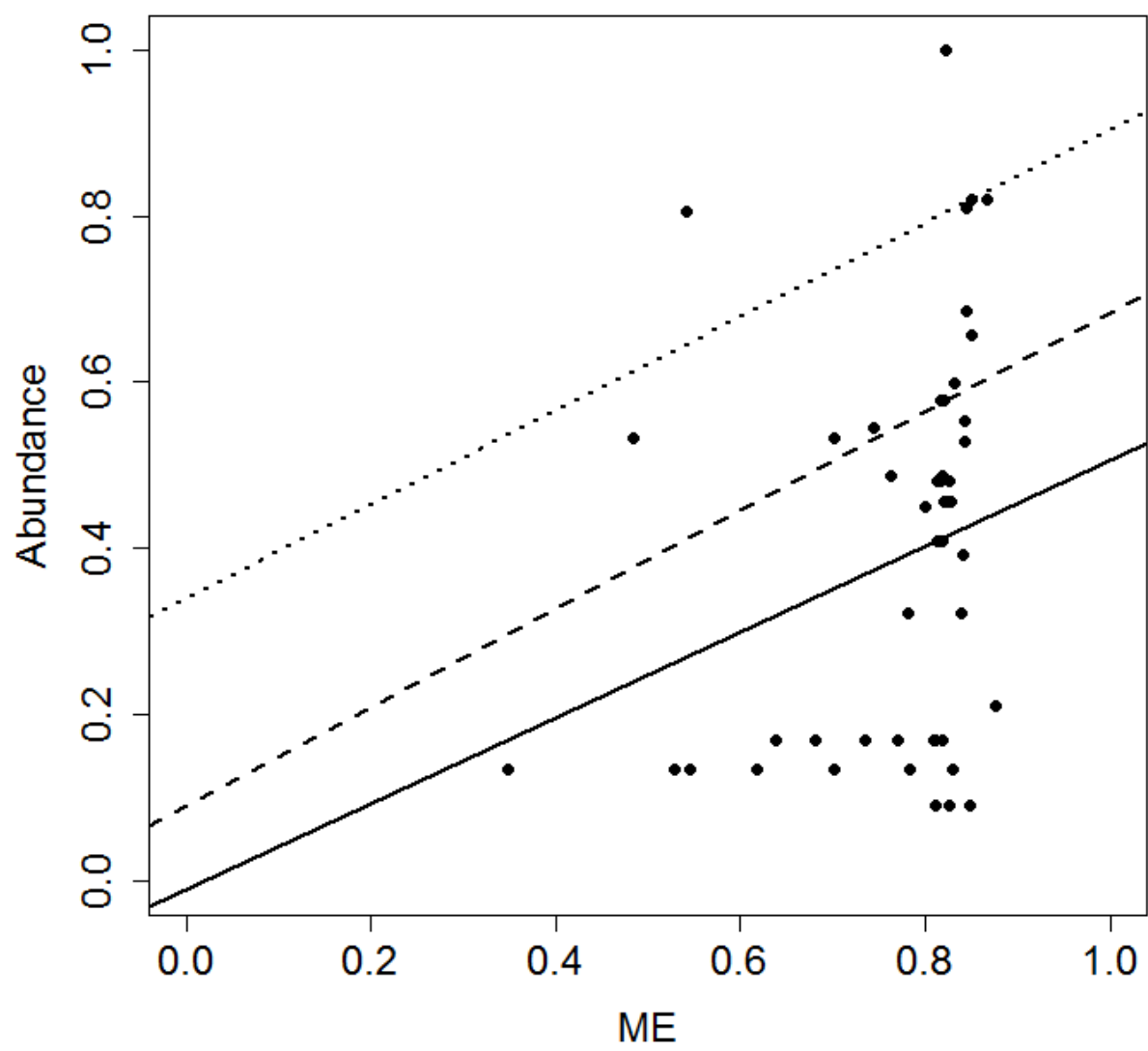

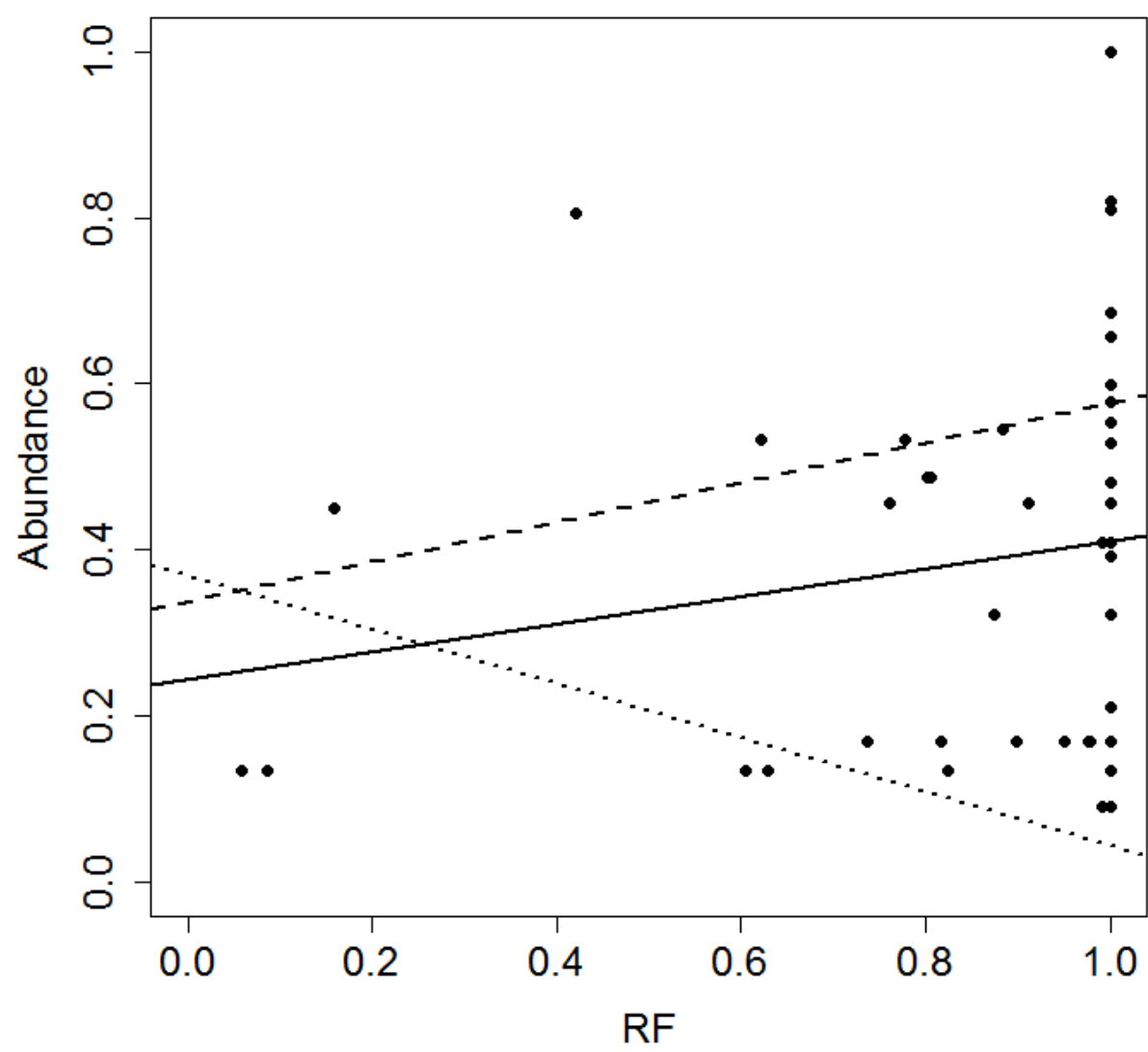

Supplement: Figure S3 — Regression lines represent the fitted relationships at different quantiles. Quantiles: solid line = 0.5; dashed line = 0.75; dotted line = 0.95.The 0.95 line often fall over the plot limits. [file peerj-04-2398-s004.pdf]
