# Supplementary material for: Patchiness of forest landscape can predict species distribution better than abundance: the case of a forest-dwelling passerine, the short-toed treecreeper, in central Italy
Source: PeerJ. 2016 Sep 8;4:e2398. doi: 10.7717/peerj.2398 (PMC5018664; doi:10.7717/peerj.2398)

Fig. S1

Artificial neural networks

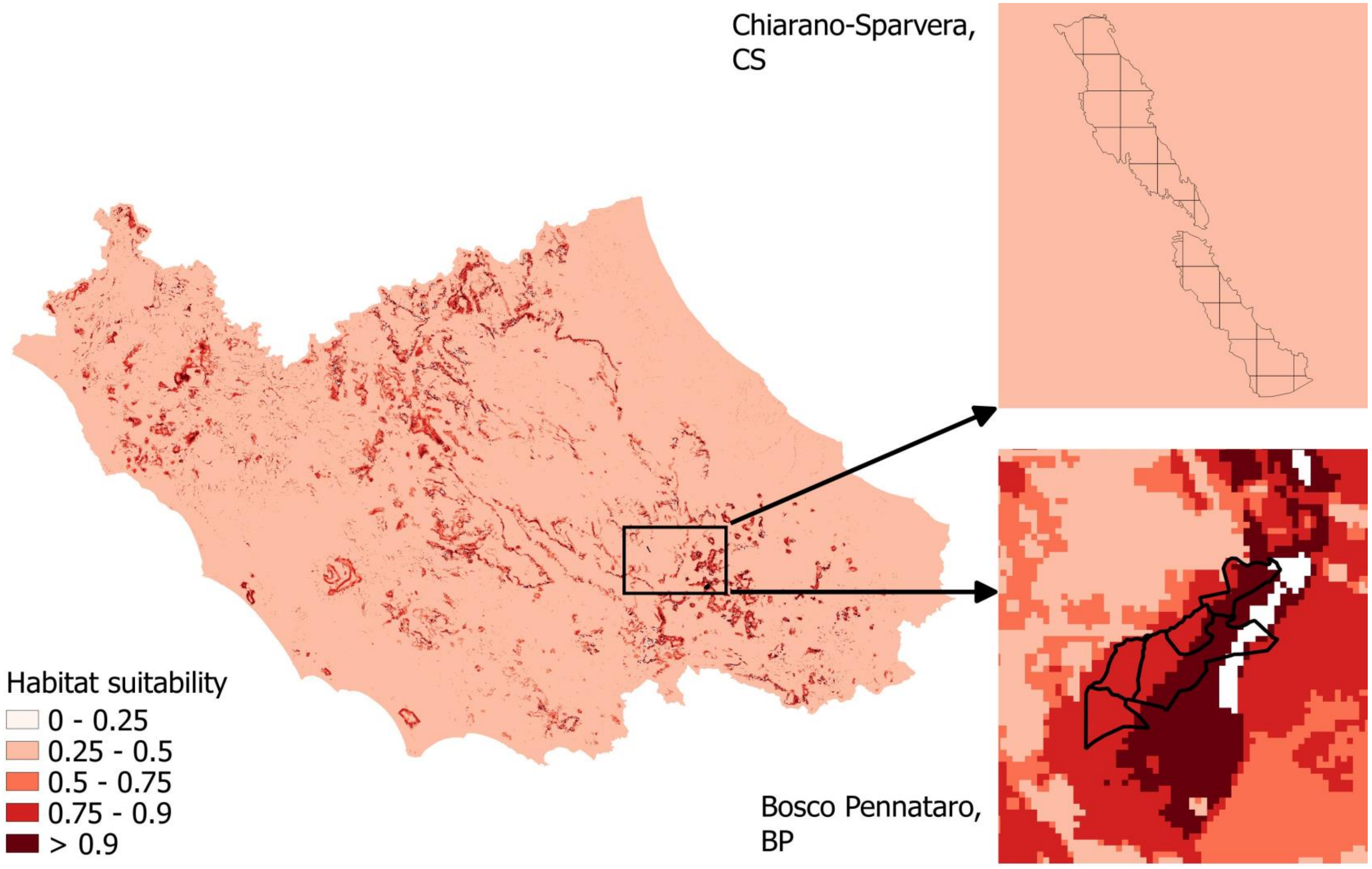

Classification tree analyses

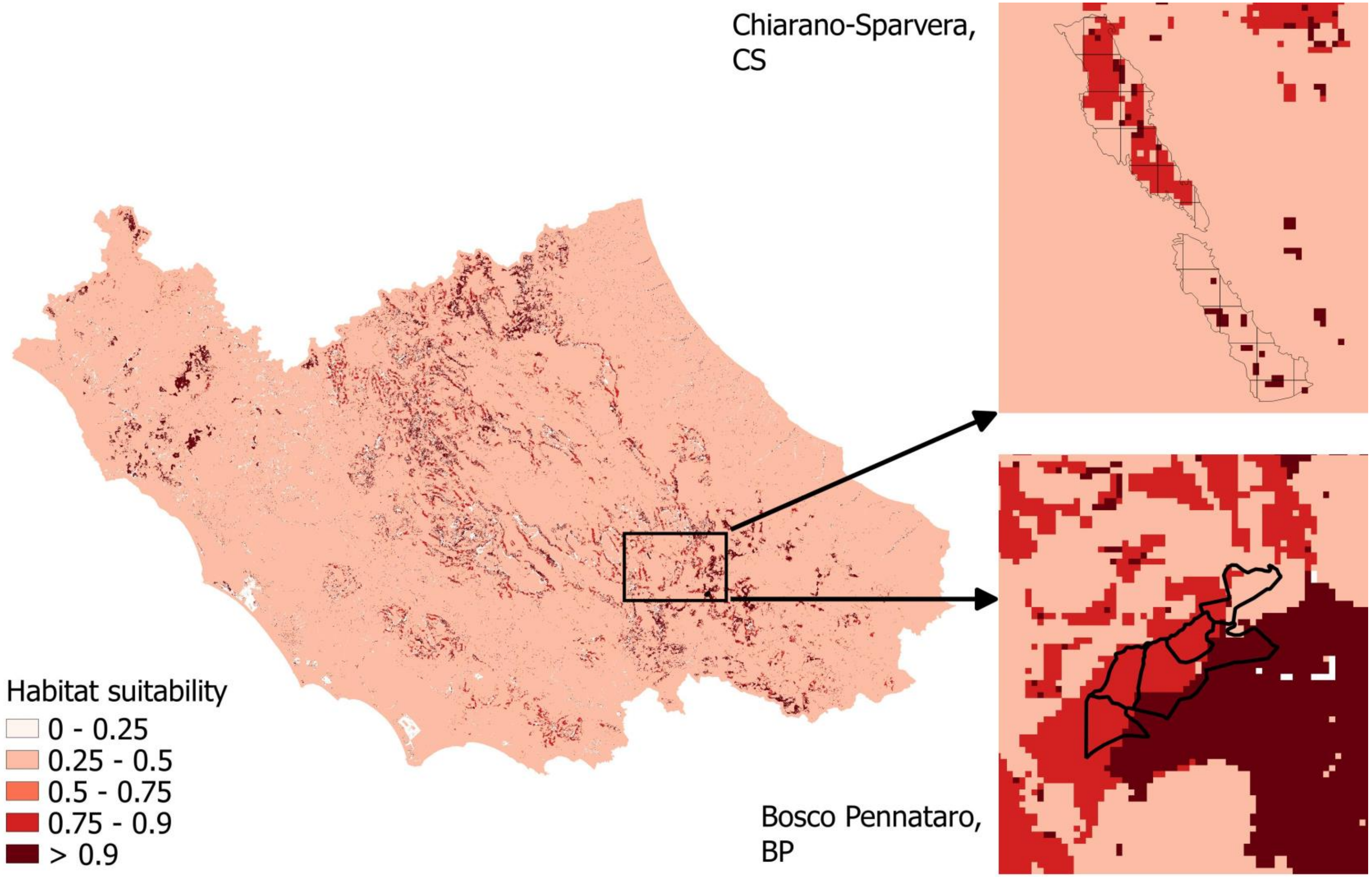

Flexible discriminant analyses

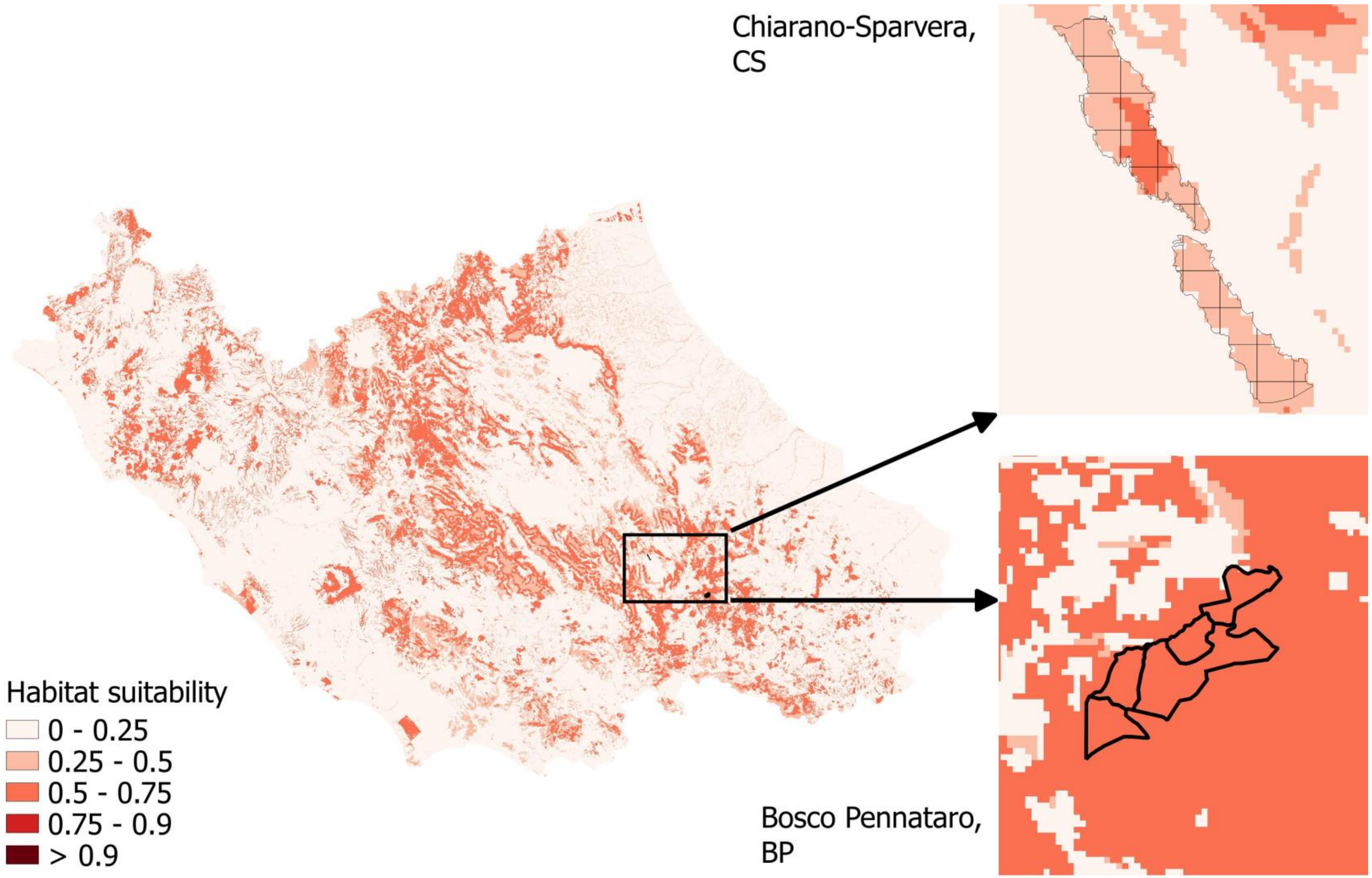

Generalized boosting models

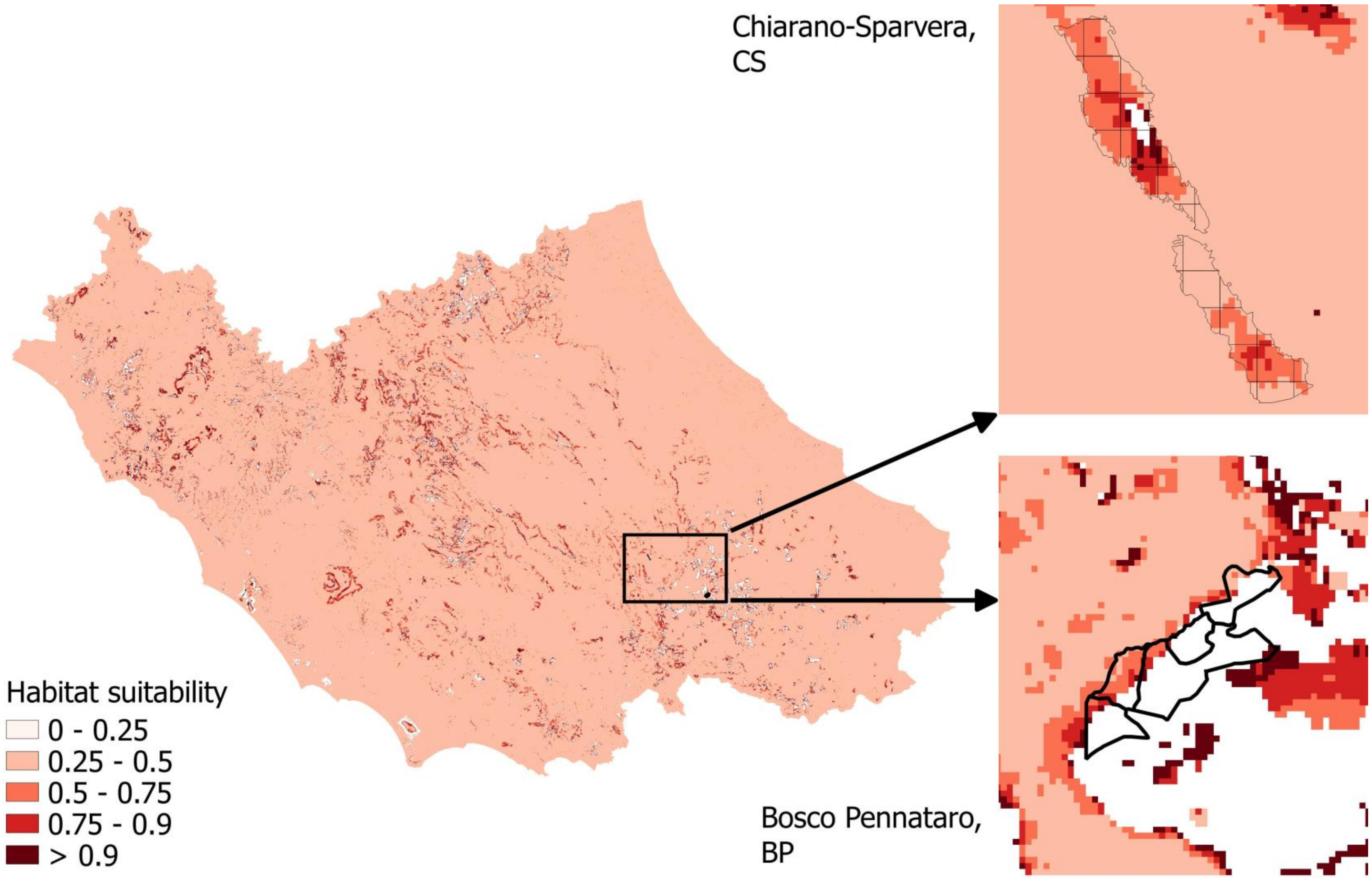

Generalized linear model

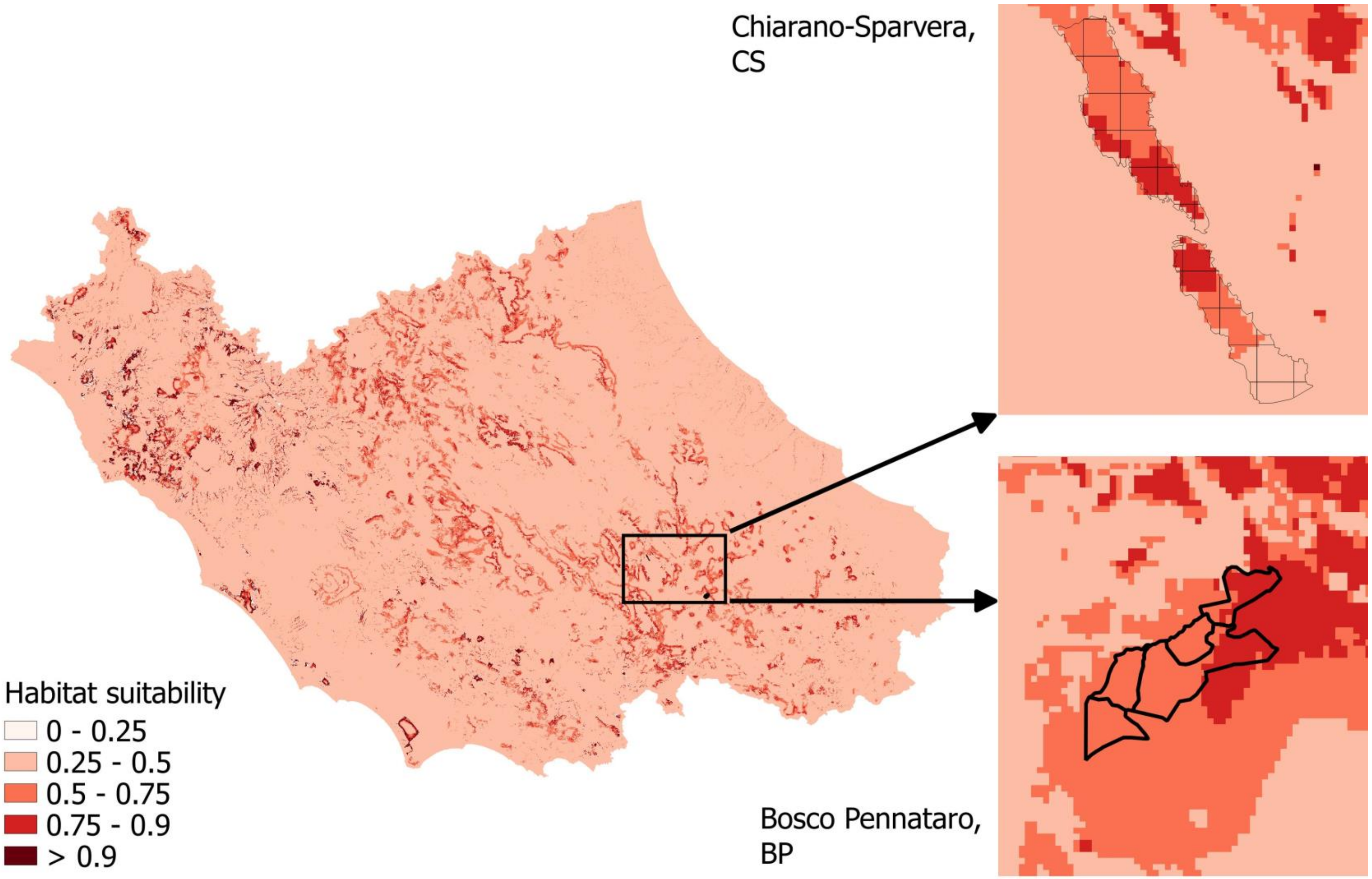

Multivariate additive regression splines

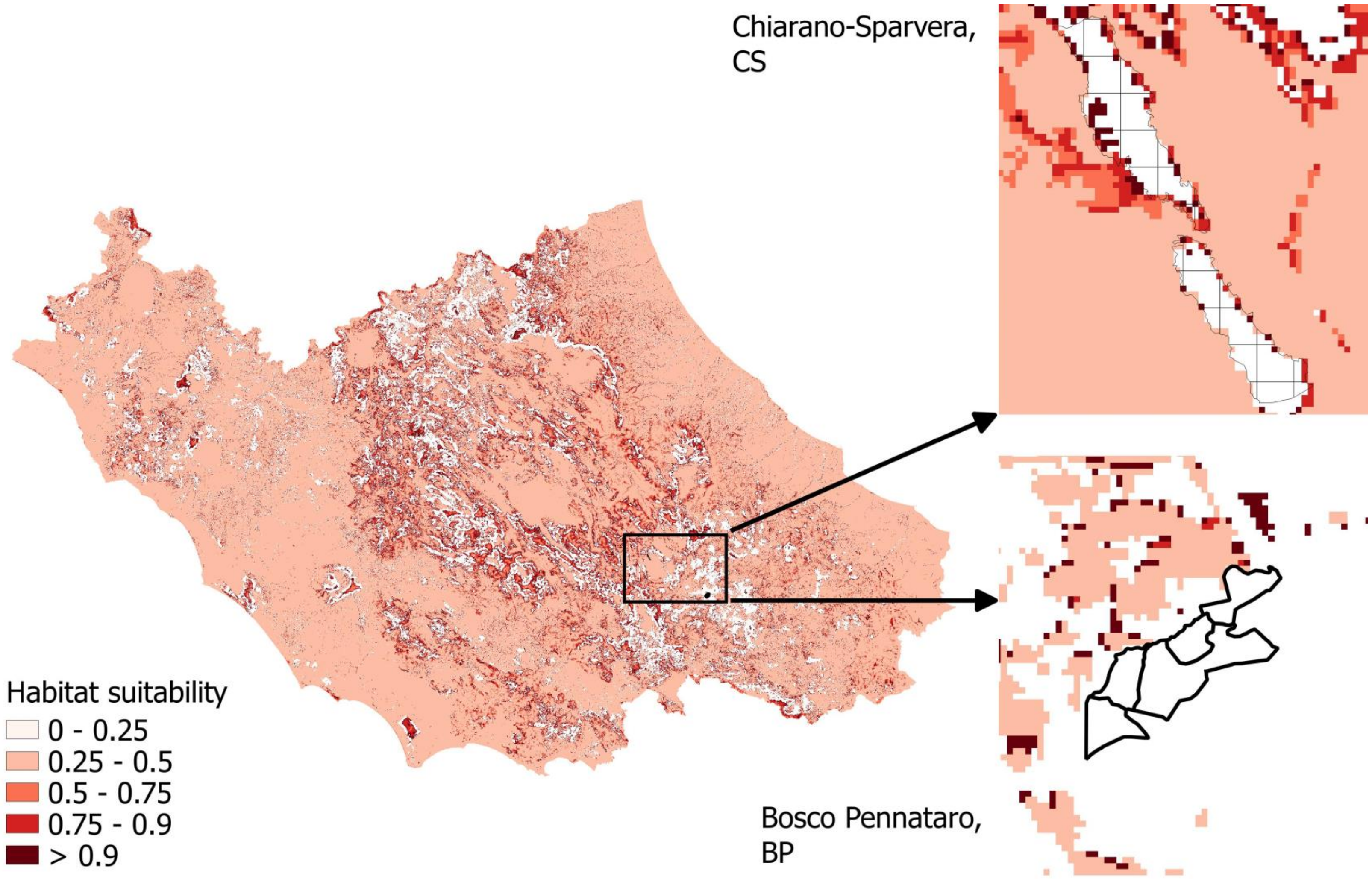

Maximum entropy

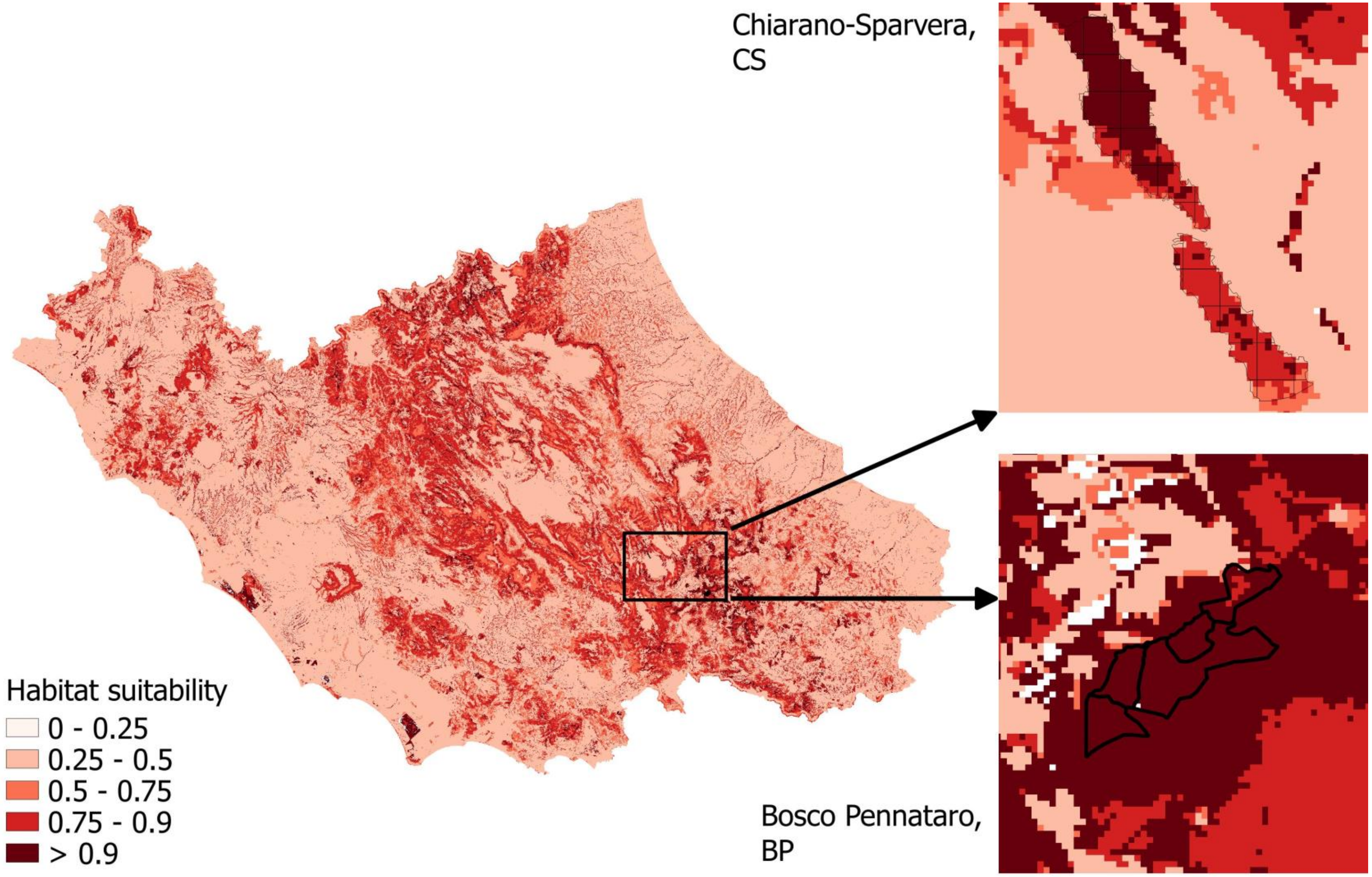

Random forests

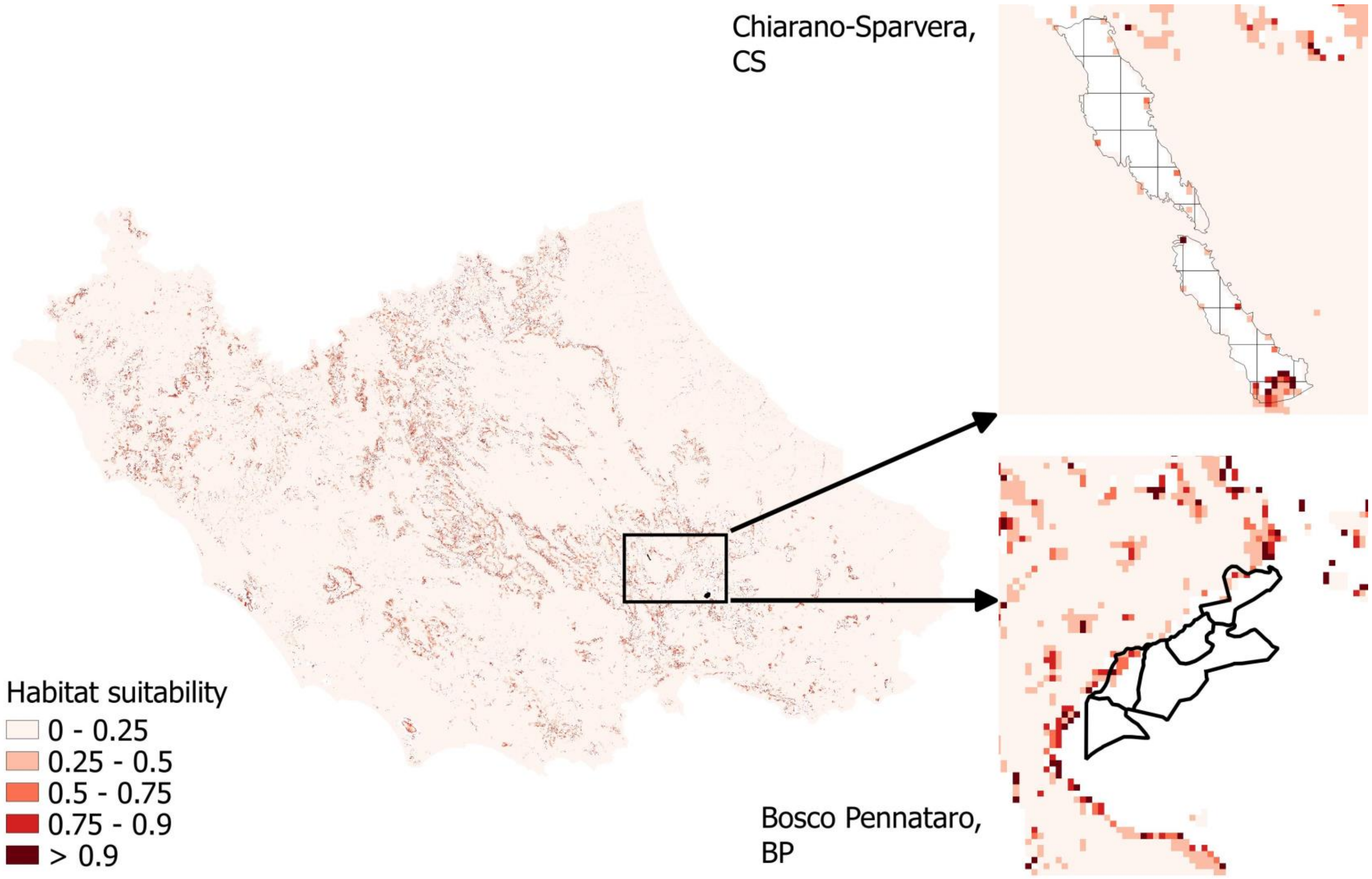

Supplement: Figure S2 — Black to white = unsuitable to suitable. [file peerj-04-2398-s003.pdf]
